# PINK: a tender X-ray beamline for X-ray emission spectroscopy

**DOI:** 10.1107/S1600577524002200

**Published:** 2024-04-25

**Authors:** Sergey Peredkov, Nilson Pereira, Daniel Grötzsch, Stefan Hendel, Dirk Wallacher, Serena DeBeer

**Affiliations:** a Department of Inorganic Spectroscopy, Max Planck Institute for Chemical Energy Conversion, Stiftstrasse 34–36, Mülheim an der Ruhr, Germany; b Berlin Laboratory for Innovative X-ray Technologies (BLiX), Institute of Optics and Atomic Physics, Technical University of Berlin, Hardenbergstrasse 36, Berlin, Germany; c Helmholtz-Zentrum Berlin für Materialien und Energie, Albert-Einstein-Strasse 15, Berlin, Germany; ESRF – The European Synchrotron, France

**Keywords:** X-ray emission spectroscopy, von Hamos dispersive spectrometers, tender X-rays, catalysis, synchrotron

## Abstract

A high-flux beamline optimized for non-resonant X-ray emission spectroscopy (XES) in the tender X-ray energy range has been constructed at the BESSY II synchrotron source. The beamline utilizes a cryogenically cooled undulator that provides X-rays over the energy range 2.1 keV to 9.5 keV. This energy range provides access to XES [and in the future X-ray absorption spectroscopy (XAS)] studies of transition metals ranging from Ti to Cu (*K*α, *K*β lines) and Zr to Ag (*L*α, *L*β), as well as light elements including P, S, Cl, K and Ca (*K*α, *K*β). The beamline can be operated in two modes. In PINK mode, a multilayer monochromator (*E*/Δ*E* ≃ 30–80) provides a high photon flux (10^14^ photons s^−1^ at 6 keV and 300 mA ring current), allowing non-resonant XES measurements of dilute substances. This mode is currently available for general user operation. X-ray absorption near-edge structure and resonant XAS techniques will be available after the second stage of the PINK commissioning, when a high monochromatic mode (*E*/Δ*E* ≃ 10000–40000) will be facilitated by a double-crystal monochromator. At present, the beamline incorporates two von Hamos spectrometers, enabling time-resolved XES experiments with time scales down to 0.1 s and the possibility of two-color XES experiments. This paper describes the optical scheme of the PINK beamline and the endstation. The design of the two von Hamos dispersive spectrometers and sample environment are discussed here in detail. To illustrate, XES spectra of phosphorus complexes, KCl, TiO_2_ and Co_3_O_4_ measured using the PINK setup are presented.

## Abstract 2

This study showcases the potential of the PINK beamline for advancing research in the field of non-resonant X-ray emission spectroscopy in the tender X-ray energy range from 2.1 keV to 9.5 keV.

## 1. Introduction

In studies of complex catalysts and metalloproteins, X-ray emission spectroscopy (XES) and X-ray absorption spectroscopy (XAS) play a major role (Bauer, 2014 ▸; Kowalska *et al.*, 2016 ▸; Castillo *et al.*, 2020 ▸; Cutsail III & DeBeer, 2022 ▸; Geoghegan *et al.*, 2022 ▸). XES of transition metals in the *K*β main line and valence-to-core (VtC) spectral regions have attracted particular interest, as these regions provide element-selective information about spin states and coordination environments, respectively.

Recent experiments showed that analysis of weak VtC spectra can help to identify ligands surrounding the probing atom (Lancaster *et al.*, 2011 ▸; Pollock *et al.*, 2013 ▸; Pollock & DeBeer, 2015 ▸; Cutsail *et al.*, 2019 ▸; Levin *et al.*, 2020 ▸). Another promising approach complements non-resonant XES with advanced resonant-based techniques that help to overcome the limitations of conventional XAS (de Groot, 2001 ▸; Szlachetko *et al.*, 2013 ▸; Lima *et al.*, 2013 ▸; Glatzel *et al.*, 2013 ▸; Castillo *et al.*, 2021 ▸). These methods are referred to as resonant XES (RXES) or resonant inelastic X-ray spectroscopy (RIXS), and involve recording an emission spectrum for each excitation energy of a regular X-ray absorption scan. Analysis of the resulting two-dimensional RXES (or RIXS plane) may allow, in certain cases, for higher-resolution XAS spectra and diminished background contributions (Hämäläinen *et al.*, 1991 ▸; Castillo *et al.*, 2017 ▸; Cutsail *et al.*, 2018 ▸). We note, however, that care must be taken in mapping RXES to XAS as there are significant deviations that may be possible depending on the transition metal multiplet structure, as well the exact RIXS plane being analyzed (*e.g.* 1*s*2*p* versus 1*s*3*p*).

XAS and XES using hard X-rays above 6 keV have become relatively routine practice today at synchrotron facilities. However, although today there are more than 60 synchrotron facilities worldwide, application for beam time is generally quite competitive and requires a proposal review process that may take up to six months or longer. During the last decade, a lot of effort has been put into the development of setups for performing XAS and XES experiments in laboratories (Malzer *et al.*, 2018 ▸; Seidler *et al.*, 2014 ▸; Błachucki *et al.*, 2019 ▸). Laboratory-based setups are now more frequently found in research laboratories, providing rapid access to the instrument. Recently, an overview of the abilities of laboratory-based XAS/XES setups was reported (Zimmermann *et al.*, 2020 ▸).

While the hard X-ray regime has become increasingly accessible at both synchrotrons and in-house laboratories, access to setups in the tender X-ray regime is limited. However, many elements with 1*s* core XES in the 2–5 keV energy region, including P, S, Cl and Ca, play important roles in both biochemistry and catalysis (Mori *et al.*, 2010 ▸; Petric *et al.*, 2015 ▸; Qureshi *et al.*, 2021 ▸). In addition, second row transition metal complexes (Mo, Ru, Rh *etc*.) actively used in photochemistry, catalysis and/or bioinorganic chemistry have also very informative *L*β, *L*γ emission lines in the 2–5 keV energy region (Levin *et al.*, 2020 ▸). Experiments conducted in the tender X-ray region can present several challenges due to higher photon absorption in media, increased radiation damage and a more pronounced scattering background. All of this imposes additional requirements on the optical design of the beamline, the vacuum system and the sample environment. Access to XES at synchrotron facilities in the tender X-ray region can be very limited and highly competitive (Mori *et al.*, 2010 ▸; Rehanek *et al.*, 2018 ▸; Butorin *et al.*, 2018 ▸; Abraham *et al.*, 2019 ▸; Rovezzi *et al.*, 2020 ▸; Qureshi *et al.*, 2021 ▸). Moreover, the availability of non-synchrotron based instruments for such experiments is also quite limited (Petric & Kavčič, 2016 ▸; Malzer *et al.*, 2018 ▸; Holden *et al.*, 2017 ▸, 2018 ▸; Abramson *et al.*, 2023 ▸).

The goal of the current project is to offer world-class capabilities for studying complex catalysts with a strong emphasis on non-resonant XES in the tender X-ray energy region in combination with high sample throughput and time-resolved (∼50–100 ms) measurements that enable *operando* investigations. Another promising technique – resonant XES, which includes VtC-enhanced XAS offering the possibility to obtain ligand-selective XAS data (Hall *et al.*, 2014 ▸; Maganas *et al.*, 2017 ▸) – will be implemented at the PINK beamline in the near future.

## 2. Beamline overview

**Figure 1:**
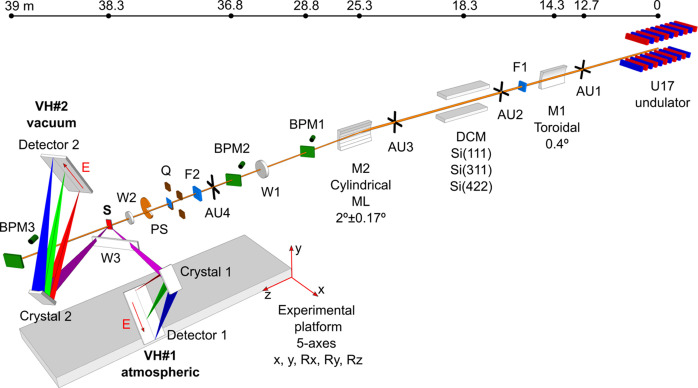
Layout of the PINK instrument showing the position (in m) of the beamline components with respect to the center of the U17 undulator. Beamline optics: toroidal (*R* = 2614923 mm, *r* = 197 mm) mirror M1, optional DCM, cylindrical (*r* = 907.5 mm) multilayer monochromator M2; motorized apertures: AU1, AU2, AU3, AU4; filters: F1 – set of water-cooled diamond attenuators (5 µm, 10 µm and 20 µm), F2 – three sets of six attenuators each (C, Al and Ta); optical beam-position monitors: BPM1, BPM2 and BPM3; Quadrant I0 monitor: Q; photon shutter: PS; vacuum windows: W1 – 12 µm Be, W2 – 10 µm diamond, W3 – 8 µm, 13 µm or 25 µm Kapton; sample: S. Atmospheric VH#1 and vacuum VH#2 von Hamos spectrometers; energy-dispersion axis: *E*.

The PINK branch was constructed in collaboration with the Helmholtz-Zentrum Berlin (HZB) on the EMIL beamline (Follath *et al.*, 2013 ▸; Hendel *et al.*, 2016 ▸) at the BESSY II synchrotron source and is depicted in Fig. 1 ▸. X-rays are provided by one of two canted undulators installed at the EMIL beamline. A planar cryogenic in-vacuum U17 undulator provides photons from 700 eV to 10 keV and the PINK branch accepts the 2.1 keV to 9.5 keV energy range (3rd to 13th harmonics). In order to optimize the branch for photon-hungry VtC XES experiments, the number of optical elements was minimized to reduce flux losses. There are adjustable slits for the beam shaping at the front of each of the optical elements.

**Figure 2:**
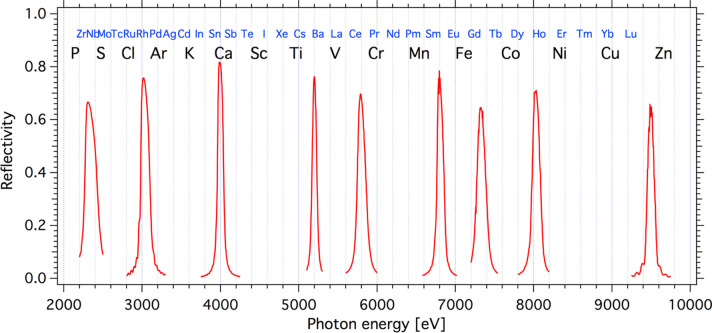
Reflectivity of the multilayer monochromator at a 2° incident angle. *K*- (black) and *L*-edges (blue) of chemical elements are depicted at the top.

The optical system has two operation modes: a primary high flux or ‘PINK’ mode with photon flux up to 10^14^ photons s^−1^ at 300 mA of the ring current serves for non-resonant XES. In the high-flux mode, the X-ray beam passes only two optical elements. A bilayer-coated (8 nm Rh top layer and 35 nm Pt bottom layer) toroidal mirror (M1) is set at 0.4° grazing incidence angle. The water-cooled M1 mirror (which is utilized for all branches that use radiation from the U17 undulator) collimates the X-ray beam vertically and focuses it horizontally at a distance of 23 m. Unfortunately, horizontal focusing capabilities of the toroidal mirror M1 do not fully correspond to the designed parameters. The theoretical horizontal focus size is ∼500 µm full width at half-maximum (FWHM), but in actual use it has a correlation with AU1 and AU3 apertures opening and varies between 500 µm and 2 mm. A set of exchangeable diamond filters (5 µm, 10 µm and 20 µm thick) is installed after M1. These filters cut low-energy radiation photons from the first undulator harmonic. This filtering action serves to reduce the power load on the downstream optical elements. The second, particularly important optical element, is a water-cooled multilayer (ML) monochromator (M2). It consists of nine vertically stacked horizontal stripes of multilayer material deposited on the three cylindrical mirrors (three stripes on each mirror). There are two kinds of multilayers: Cr/B_4_C and W/B_4_C. In the high-flux mode, M2 is operated at a fixed grazing incident angle of 2° and performs three tasks. The first is switching of the X-ray beam into the PINK branch. The second is focusing the beam vertically at the sample position. Due to the slightly different quality of the substrates measured, the vertical focus size varies from the theoretical to the measured values of 25 µm to 35 µm (FWHM). The third function of the M2 is photon-energy selection. Reflectivity of individual stripes as a function of the photon energy for a 2° angle of incidence is plotted in Fig. 2 ▸. At this angle, the ML monochromator can pass photons with nine fixed energies. Each stripe corresponds to one photon energy (see Table 1 ▸). The photon bandwidth of the multilayer stripes varies from 65 eV to 155 eV (FWHM) and almost the full photon flux of a single undulator harmonics can pass the monochromator generating the so-called ‘PINK beam’.

**Figure 3:**
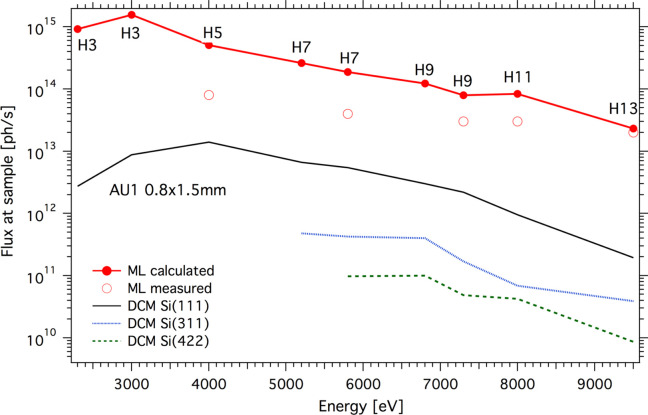
Calculated (red solid circles) and measured (red open circles) photon flux at the sample position for the high-flux (PINK) mode using a multilayer monochromator. Flux values are calculated and measured with the front aperture AU1 opened to 0.8 mm × 1.5 mm. The symbols H*n* indicate the undulator harmonics used, where *n* is a harmonic number. The estimated flux in high-monochromatization mode with a DCM inserted is also shown.

The PINK photon flux at the sample position is reported in Fig. 3 ▸. For the flux measurements, a Hamamatsu Si diode was inserted into a direct beam attenuated by F2 filters. At higher energies the measured flux agrees with the values calculated using the *xrt* code (Klementiev & Chernikov, 2014 ▸). The discrepancy increases at lower photon energies. This can be associated with the fact that flux measurements at energies below 5–6 keV become more sensitive to the thicknesses of vacuum windows and absorbing foils. Additionally, at lower energies, we have to reduce the AU3 horizontal aperture size in order to decrease the power on the ML to avoid overheating of the mirror and shape the beam into the designed horizontal size of 500 µm (FWHM).

Experiments involving RXES/XAS techniques can be done in a high monochromatic mode with reduced photon flux in which the DCM monochromator is inserted into the beam. This second operation mode is under commissioning at present. The outgoing monochromatic beam is 20 mm higher than a PINK beam and hence we have to raise the ML monochromator as well as the endstation. Since the monochromatic beam also passes the ML monochromators, its energy has to be set to the region needed for RXES/XANES measurement. This can be done by changing the angle of incidence of the ML monochromator. By varying the M2 angle in the range 2° ± 0.15°, we can shift the energy of the ML monochromator by approximately ±500 eV, thus nine multilayer stripes cover almost the full 2.1 keV to 9.5 keV energy range. The beamline vacuum tubing/apertures accept ±0.17° horizontal deflection of the beam. The maximum horizontal beam displacement at the sample position is ±80 mm. The endstation is situated on a movable motorized platform and connected to the beamline via flexible bellows that allow it to be adjusted to the beam height. Any measurements involving the DCM are done at a fixed M2 angle; no ML monochromator scanning is allowed, and the photon flux impinging on the sample is a product of convolution of the monochromatic beam with a multilayer mirror reflectivity curve.

## 3. Experimental station

### 3.1. Vacuum system

Using tender X-rays places additional constraints on the vacuum system. The PINK setup has three isolated areas. A 12 µm-thick and 7 mm-diameter Be window braised into a CF40 flange manufactured by Materion protects the ultra-high-vacuum part of the beamline and the storage ring. An in-house-made 10 µm CVD diamond vacuum window (6 mm diameter) separates 10^−6^ mbar vacuum of the diagnostic chamber incorporated into the endstation and a sample environment area operated between 10^−6^ mbar and 10 mbar pressure. When experiments are run under He atmosphere at ambient pressure, an additional self-made 25 µm Kapton window is installed downstream of the diamond window.

### 3.2. Beam diagnostic

Three beam-position monitors (BPMs) are installed for X-ray beam alignment: 3.5 m downstream of M2 (BPM1), 2 m before (BPM2) and 0.7 m after (BPM3) the sample position (as shown in Fig. 1 ▸). The BPMs are equipped with motorized 100 µm-thick YAG and 20 µm-thick CVD diamond screens rotated through 45° about a vertical axis to the beam propagation direction and monitored by high-resolution video cameras. A 260 mm-long diagnostic chamber comprises a set of attenuators, I0 monitor and a photon shutter (F2, Q and PS in Fig. 1 ▸). Three linear piezo-positioners carry a diode, two apertures (500 µm and 30 µm in diameter) and three sets of CVD diamond, Al and Ta foils of different thicknesses that can be combined for attenuation optimization. The transmission coefficient can be set from 0.9 to 0.0001 over the whole energy range. The X-ray exposure of the sample is controlled by a vacuum fast beam shutter (PS) Uniblitz XRSR6 that opens only at the acquisition time to prevent continuous exposure of the sample. The shutter has a large 6 mm aperture and an opening/closing time of ∼20 ms. The maximum operating frequency is 2 Hz.

The intensity and beam-position monitor I0 uses an array of four 10 mm × 10 mm PIN photodiodes (S3590-09 Hamamatsu) that detect radiation scattered from a foil in backscattering radiation geometry (Tono *et al.*, 2011 ▸). There are three low-*Z*-material foils fixed on a linear piezo stage: 10 µm CVD diamond, 2 µm Si_3_N_4_ and 25 µm Kapton.

### 3.3. Dispersive spectrometer

High incoming photon flux and excessive radiation sensitivity of biomolecules determined the choice of the analyzer geometry. Dispersive von Hamos spectrometers detect X-rays of a range of energies simultaneously (van Hámos, 1933 ▸; Vane *et al.*, 1988 ▸; Hoszowska *et al.*, 1996 ▸). They are suitable for time-resolved measurements and are actively used at free-electron laser facilities (Alonso-Mori *et al.*, 2012 ▸; Szlachetko *et al.*, 2017 ▸; Canton *et al.*, 2023 ▸). Nowadays, von Hamos spectrometers are becoming increasingly popular at synchrotrons and laboratories (Malzer *et al.*, 2018 ▸; Kalinko *et al.*, 2020 ▸; Zimmermann *et al.*, 2020 ▸). The PINK setup is equipped with two dispersive von Hamos analyzers that can be operated simultaneously. The current design achieves unique two-color XES collection capabilities that offer scope for more detailed investigation of catalysts and metalloenzymes with more than one metal at the active sites. Designing the spectrometers, we aimed to strike a balance between resolution, efficiency, the space available for the sample environment and cost. Our main concern at first was to obtain the highest efficiency while maintiaining a moderate energy resolution because the natural width of *K*β, *L*β and VtC lines is usually larger than 1–1.5 eV.

A von Hamos spectrometer resolution is mainly affected by (1) the X-ray beam spot size at the sample position, (2) the analyzer crystal radius of curvature and diffraction angle, (3) the sample surface orientation relative to the dispersive crystal and the X-ray penetration depth into the sample, and (4) the detector pixel size. The small vertical size of the PINK beam (30 µm FWHM) offers an advantage to an analyzer with energy dispersion in the vertical direction. Higher resolution can be achieved by increasing the bending radius, but a price must be paid in the loss of efficiency. Building a large crystal array can increase the solid angle of collection (Alonso-Mori *et al.*, 2012 ▸; Kalinko *et al.*, 2020 ▸). The downside of this approach is a dramatic increase of costs and the extra space required. Thus, we have prioritized a single-crystal solution.

We did not investigate cylindrically bent crystals with very short radii (*R* ≤ 250 mm) because using them significantly limits room for a sample environment. That aside, manufacturing of bent short-radius crystals without elastic deformations – which can significantly reduce their intrinsic resolution – is extremely challenging. Striping of the crystal is an alternative way of manufacturing short-working-distance crystals (Szlachetko *et al.*, 2012 ▸, 2017 ▸). Here the cylindrical surface is formed from an array of small flat stripes, but manufacturing capabilities and the quality of such crystals are also very limited. A strip bent crystal also has a larger focus size. A bent crystal focuses the source width to detector down to 1:1. A striped crystal forms an image on the detector with a width in the focusing direction that is twice the stripe width.

**Figure 4:**
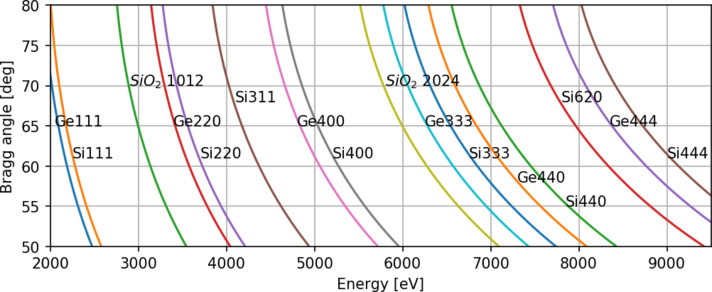
Energy ranges available for the nine-crystal library at PINK: Si(111), Ge(111), Quartz (1012), Si(110), Ge(110), Si(311), Si(100), Ge(100) and Si(310). The majority of the 2 keV to 9.5 keV energy range can be covered within 80° to 50° Bragg angles.

Optimization of the spectrometer geometry was done using *xrt* ray-tracing software (https://xrt.readthedocs.io). Ray tracing was performed for a collection of nine crystals that efficiently covered the 2 keV to 9.5 keV energy range (see Fig. 4 ▸). Curvature radii of 500 mm, 350 mm and 250 mm were chosen for the simulation. We also considered both bent and striped crystals. The ray-tracing simulations for ideal bent crystals of *R* = 250 mm showed that, for a beam size of 30 µm × 500 µm (FWHM, V × H) and Bragg angles in the range 80° to 50°, resolution varies between 0.2 eV and 0.4 eV, and for a 1 mm strip bent crystal between 0.2 eV and 0.8 eV. For larger radii, the resolution varies between 0.1 eV and 0.3 eV. Both PINK spectrometers are designed to accept *R* = 250 mm and *R* = 350 mm analyzer crystals. Currently only shorter radius *R* = 250 mm crystals are available. The bent crystals were produced by Bourevestnik (St Petersburg, Russia). The strip bent crystals with a segment width of 1 mm were manufactured in collaboration with the Paul Scherrer Institute (PSI). The analyzer crystal dimension in the focusing (horizontal) direction is 100 mm and in the dispersive direction varies from 25 mm to 50 mm depending on the crystal. The specifications of both spectrometers are presented in Table 2 ▸.

#### 3.3.1. Atmospheric von Hamos spectrometer

The atmospheric von Hamos spectrometer is designed for measurements above 6 keV (VH#1 on Fig. 1 ▸). The analyzer crystal is oriented at 90° with respect to the X-ray propagation direction in order to reduce the elastic scattering background and at 45° to the sample surface. The analyzer focuses X-rays in the horizontal direction and spatially separates different energies in the vertical direction. The spectrometer is built on the constant exit-direction scheme where the Bragg angle of a dispersive crystal is changed by the crystal rotation. In this context, ‘constant exit direction’ denotes the consistent position of the crystal relative to the sample. This is in contrast to a classical von Hamos scheme where linear translation is used. In the geometry used at the PINK endstation, the center of the dispersive crystal constantly stays in the horizontal plane passing through the incoming X-ray beam, and the source of X-ray emission and the analyzer crystal always stay on the same axis. This has two advantages. Independent of the crystal Bragg angle, the maximum vertical opening needed to illuminate the 50 mm-high crystal is only 11.4°, which allows the vertical height of the exit window on the sample chamber to be minimized. Additionally, in this configuration, the spectrometer is effectively insensitive to sample thickness or propagation depth of the X-ray beam into the sample (which increases with higher incoming X-ray energies).

**Figure 5:**
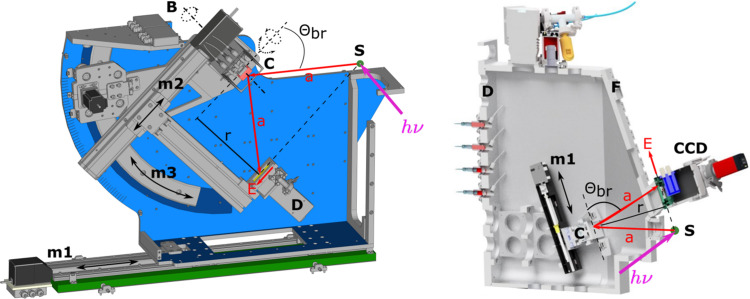
View of the PINK atmospheric von Hamos spectrometer with a fixed-exit direction VH#1 (left) and vacuum von Hamos spectrometer VH#2 (right), where *E* is the energy dispersion axis. S – sample; C – analyzer crystal; D – detector; m1, m2 and m3 – motorized axes; F1 – exchangable flange; F2 – flange with hinges; *R* – rotation axis of the crystal.

The mechanical implementation of the spectrometer is shown in Fig. 5 ▸ (left). The crystal (C) and the detector (D) are mechanically parallel at the distance *r* = 250 mm. The sample(S)-to-crystal distance *a* = 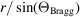 is adjusted by the linear stage m1, moving the whole spectrometer as a unit. The angle of incidence of the X-rays onto the crystal is adjusted by means of a curved motorized rail segment (m3) where (*R*) is the axis of rotation. The same curved segment also rotates the linear stage m2 carrying the detector. A crystal-to-detector distance is set by the linear stage m2. Final adjustment of the dispersive image on the detector is done with a pair of piezo-stages rotating the crystal (yaw and roll).

Two detectors are currently available. The first is a 2D Eiger2 R 500K (75 µm × 7 5 µm pixel size, 512 × 1030 pixels). The second detector is a 1D Mythen2 1K X (50 µm × 8 mm stripe size, 1280 stripes). The advantage of using a 2D detector is easier alignment of the crystal by controlling the focus size. The design of the spectrometer allows Bragg angles between 82° and 45° to be reached. The upper Bragg angle is limited by the sample environment chamber dimensions. The typical energy window visible by the 80 mm-long Eiger detector is 300 eV to 500 eV. The average solid angle collected by the crystal and imaged by the detector is ∼0.05 sr and (0.9–1.5) × 10^−4^ sr eV^−1^ depending on the span of Bragg angles. For crystals with *R* = 250 mm, the X-ray path in air between the sample and the detector is approximately 500–540 mm. At 7 keV the air transmission remains above 40%. Therefore, the use of an He bag, which is commonly employed to minimize X-ray absorption in the air for spectrometers with bending radii of 0.5 m to 1 m, is not strictly necessary. This makes operating the spectrometer more convenient but at the cost of reduced signal.

#### 3.3.2. Vacuum von Hamos spectrometer

The vacuum von Hamos spectrometer is designed for measurements between 2 keV and 6 keV and has a working pressure of ∼10^−5^ mbar. The vacuum spectrometer is fixed at 45° to the X-ray propagation direction and perpendicular to a sample surface (VH#2 in Fig. 1 ▸). The detector is located above the sample.

A fixed-exit direction scheme has a number of advantages over a conventional von Hamos design (see Section 3.3.1) but its engineering implementation in the case of a vacuum spectrometer is challenging. It requires a large vacuum chamber with an in-vacuum detector, water cooling for the detector and a number of motion stages. We implemented a classical von Hamos scheme but tilted 20° about the vertical axis (see Fig. 5 ▸, right). When the center of the dispersive crystal (C) is in the incident beam, the horizontal plane of emitted X-rays from a sample (S) illuminates the center of the crystal at a 70° Bragg angle. An analyzer crystal is mounted on an in-vacuum linear stage m1 inside the vacuum chamber. By moving the crystal along the m1 axis the Bragg angle can be changed by 70 ± 11°. In this case, the dispersive crystal is only slightly out of the horizontal plane and mimics the fixed-exit direction scheme. There is a pair of motorized blades in front of the crystal. These blades help to mask unused crystal surface in order to reduce diffuse scattering background. The upper blade carries a diode measuring the total fluorescence yield (TFY). This signal is also used to monitor synchronization of the fast shutter (Fig. 1 ▸, PS) and the CCD.

A windowless GreatEyes CCD detector (256 × 1024 pixels, 26 µm × 26 µm pixel size) is mounted on an exchangeable vacuum flange (F1) to mechanically guarantee the detector position and parallelism of the crystal and the detector (D). The field of view of the 26.6 mm-long CCD camera varies from 20 eV to 80 eV depending on the energy and Bragg angle.

Switching between XES measurements for different emission lines takes about 1.5–2 h. It requires venting of the spectrometer chamber, changing a dispersive crystal and mounting the detector to a new position. To make this procedure easier, the crystal mount and all electrical feedthroughs are fixed to a door with hinges and quick clamps (F2). We have a collection of seven exchangeable flanges (F1) and each flange has 2–3 pre-cut windows for the CCD mounting.

For the 2300–2500 eV and 5400–5500 eV energy ranges there is no available Si, Ge or quartz crystal with working Bragg angles larger than 60°. XES measurements of S *K*β, Mo *L*β and V *K*β lines demand operation of the vacuum spectrometer at 50° to 57° Bragg angles. Especially for these measurements, we manufactured a second vacuum chamber for a von Hamos spectrometer tilted by 40°. It covers Bragg angles of 50° ± 11°. The chambers use the same base frame and can be easily exchanged by a small crane.

#### 3.3.3. Energy calibration

**Figure 6:**
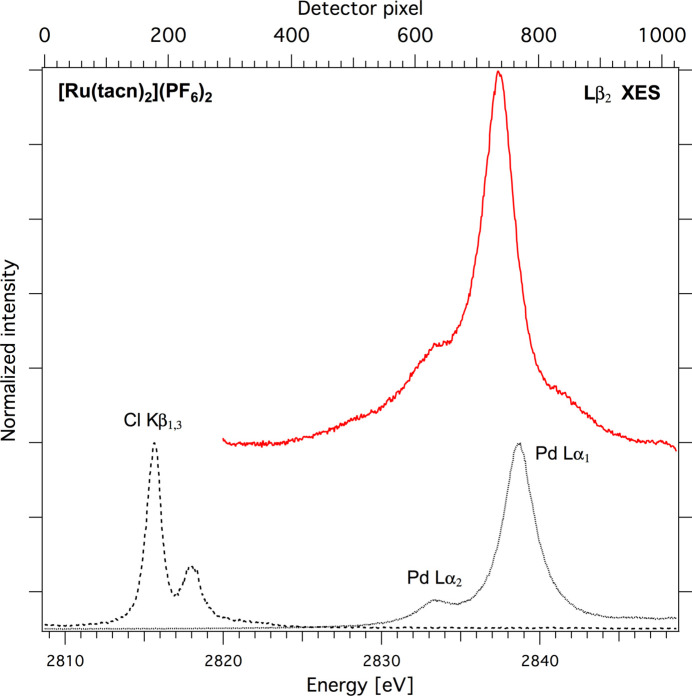
Ru 4*d*-to-2*p* XES spectrum (top) and reference spectrum (bottom) used for the energy calibration procedure. The [Ru(tacn)_2_](PF_6_)_2_ spectrum was adapted from the work by Levin *et al.* (2020 ▸),

The most commonly used energy calibration procedure of an emission spectrometer applied at a synchrotron facility employs monochromatic light. Monochromatic X-rays provided by a monochromator were elastically scattered from a light-*Z* target placed at the sample position and recorded by an analyzer which gives a precise energy calibration. At the PINK beamline in high-flux mode there is no access to monochromatic light and, as an alternative, emission lines from reference samples are used for the calibration procedure. An example of an 4*d*-to-2*p* XES spectrum of the Ru complex is shown in Fig. 6 ▸. This measurement was done with the vacuum von Hamos spectrometer using an SiO_2_(1012) crystal (*d* = 2.282 Å) with *R* = 250 mm (Levin *et al.*, 2020 ▸). In the present geometry, a 1 inch-long CCD detector can capture an approximately 50 eV-wide energy window. For energy calibration, KCl powder and Pd foil samples were used. To establish the energy scale, the positions of the Cl *K*β_1_, Pd *L*α_2_ and *L*α_1_ emission lines were picked by fitting both reference spectra with two Voigt profiles. Corresponding energies of 2815.6 eV, 2833.29 eV and 2838.61 eV for these three peaks were taken from the work of Thompson & Vaughan (2009 ▸) and Liu *et al.* (2004 ▸) and converted to Bragg angles,

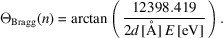

For a von Hamos spectrometer, geometry X-rays diffracted at the Bragg angle Θ_Bragg_ arrive at the detector at a position described by

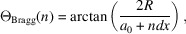

where *n* is the detector pixel number, *dx* is the pixel size, *a*_0_ is the distance from the sample to the first pixel on the detector and *R* is the curvature radius of the crystal. By fitting this function with two free parameters *R* and *a*_0_, a one-to-one correspondence between the Bragg angle and the detector pixel number is obtained. By converting the Bragg angles to corresponding energies, we obtain the energy calibration *E*(*n*) of the spectrometer. This function is quasilinear for a 1 inch CCD detector, but the 80 mm-long Eiger chip already shows substantial *E*(*n*) nonlinearity. This energy calibration procedure has to be repeated after any realignment of the beamline or analyzer optics, or after sample exchange.

Reproducibility of the energy calibration process relies mainly on the accuracy of the peak-fitting procedure applied to reference spectra. In day to day measurements, the reproducibility is generally within a single detector pixel and is typically less than ±30 meV. We repeated K *K*β main line XES measurements performed on KCl, as outlined in Section 4 (see Fig. 13), with a two-year interval after the beamline upgrade and vacuum spectrometer rearrangement. Energy calibration was achieved using Sb *L*α_1_ (3604.72 eV) and *L*α_2_ (3595.32 eV) lines measured at the Sb foil. The energy of the *K*β_13_ peak exhibited variations within the range ±50 meV.

### 3.4. Sample environment

Radiation damage is an important consideration for XES/XAS studies of many molecular complexes and metalloproteins (Van Schooneveld & DeBeer, 2015 ▸). The PINK beamline delivers a high photon flux, particularly in the tender X-ray energy range. This elevated photon flux amplifies the acquisition of valid signals but concurrently escalates the rate of radiation-induced damage. Consequently, the damage rate becomes notably pronounced at lower photon energies within the range 2 keV to 5 keV, primarily due to the reduction in the attenuation length of the material. There are three primary ways to measure radiation-sensitive specimens: scanning of the sample, using high flow rate liquid cells and subjecting samples to cryogenic temperatures (Meents *et al.*, 2010 ▸). We have incorporated all three of these approaches into our experimental protocol. There are two sample chambers that can be incorporated into the PINK vacuum system. Both chambers as well as the samples inside are oriented at 45° to the incident beam.

#### 3.4.1. LiquidSEC

**Figure 7:**
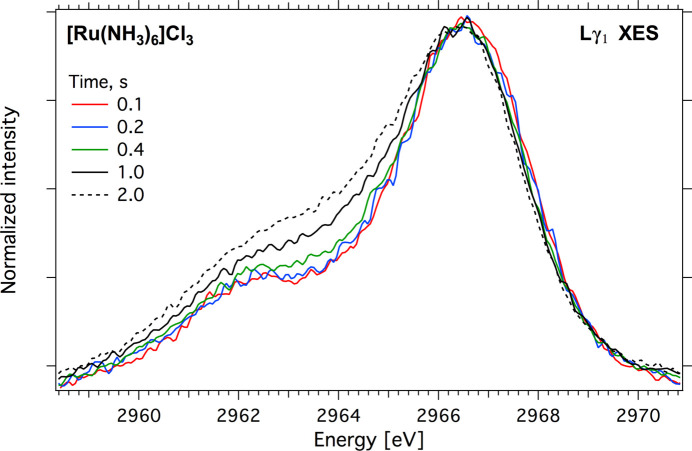
Radiation damage of [Ru(NH_3_)_6_]Cl_3_ under 10^13^ photons s^−1^ photon flux. The instantaneous irradiation time during one scan over the sample area is 0.1 s. The measurement time for one pass is 4 min.

A liquid sample environment chamber (LiquidSEC) is available for room-temperature (RT) experiments, which operates under vacuum conditions of 2 mbar to 10 mbar or an He atmosphere up to 1 bar. The vertical dimension of the chamber is deliberately limited to 10 cm to create additional room for both detectors, thereby facilitating measurements at Bragg angles of up to ∼82°. Two linear piezo-stages provide fast (up to 2 mm s^−1^) vertical and horizontal positioning of a sample carrier that can hold four solid samples and a liquid-flow cell or electrochemical cell. A miniature camera installed inside the chamber is used for alignment and control of operating the flow/electrochemical cells. The chamber has two exit windows for fluorescent radiation: a 13 µm-thick Kapton window (31 mm × 18 mm) oriented towards the atmospheric von Hamos spectrometer (VH#1 on Fig. 1 ▸) and an 8 µm-thick Kapton window (18 mm × 18 mm) that separates the sample environment chamber and the second vacuum von Hamos spectrometer (VH#2 on Fig. 1 ▸). The later window enables sample exchange without venting the vacuum spectrometer. The chamber has two feedthroughs for liquid supplies and a DSUB-9 socket for connecting a potentiostat and an LED light source for studying photoinduced reactions. KF40 flanges are used to connect the sample chamber to the diagnostic chamber from the upstream side and to the BPM3 chamber on the downstream side. Continuous scanning of a sample irradiation area at high speeds (500–1000 µm s^−1^) helps to reduce the radiation dose at a given a spot. By varying the scanning speed, we can reduce the instantaneous irradiation time down to 100 ms to 50 ms during one scan over the sample area. An example of radiation damage of the [Ru(NH_3_)_6_]Cl_3_ complex under an incoming photon flux of 10^13^ photons s^−1^ is shown in Fig. 7 ▸. A 64 mm^2^ powder sample was scanned with 500 µm s^−1^ velocity that is equivalent to 100 ms radiation at the spot. The measurement was repeated ten times with increasing radiation dose. The *L*γ_1_ XES spectra presented show no changes between 0.1 s and 0.2 s radiation time. The first changes appeared around 2963 eV after ∼0.4 s and became more pronounced after 1 s. The conventional scanning spectrometers also allow fast sample motion, but analysis of the spectra requires additional normalization by the total fluorescence signal, which can be challenging.

#### 3.4.2. CryoSEC

Cryogenic conditions for XES/XAS measurements are highly desirable at synchrotron facilities, as the low temperatures help to reduce the radiation damage of the samples, stabilize reactive complexes, prevent decomposition of intermediates and enable preparation of frozen solutions. The highly intense PINK beam bears up to several watts of energy onto the 30 µm × 500 µm spot. For example, at 3 keV in water, 85% of the X-ray photons are absorbed within 100 µm. Powder and biological samples typically have poor thermal conductivity. To efficiently cool down samples and avoid hot spots, an He exchange gas must be used.

**Figure 8:**
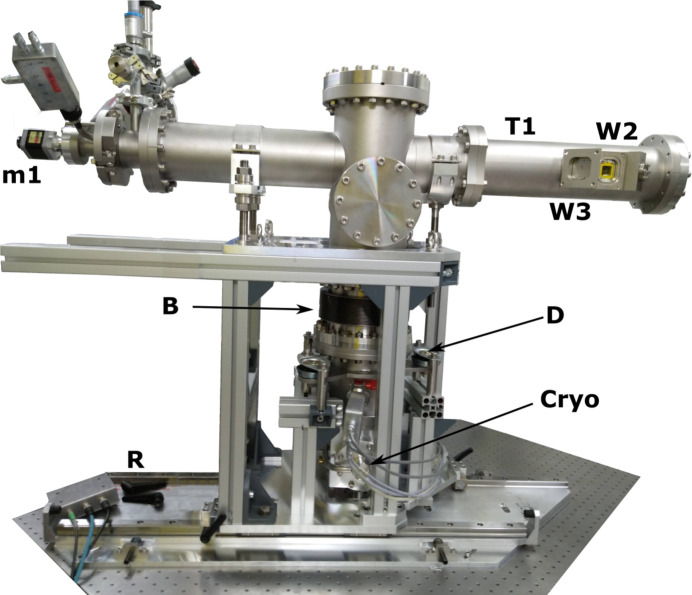
View of the cryogenic sample environment chamber CryoSEC. T1 – vacuum chamber; Kapton vacuum windows: W2 – 8 µm, W3 – 25 µm; m1 – motor for horizontal motion of the sample carrier; R – THK rails; Cryo – closed-cycle cryocooler; B – bellow; D – vibration dumper.

A cryogenic sample environment chamber (CryoSEC) was designed entirely by the sample environment group of HZB (see Figs. 8 ▸ and 9 ▸). The CryoSEC consists of an internal cold sample chamber (T2) that can be operated under 10 mbar to 1000 mbar He pressure and outer insulation vacuum chambers (T1). The insulation chamber is connected to the diagnostic chamber on the front side and to the BPM3 chamber on the back side, and maintained at 10^−5^ mbar pressure. To transfer heat from the sample to a cold Cu exchange tube, He gas at a pressure of 5–20 mbar is used. This tube is connected to the cold finger of the cryocooler via a Cu braid. The Sumitomo RDK-500B one-stage closed-cycle He cryocooler (Cryo) is situated at the bottom of the chamber and has a cooling capacity of 40 W. It can cool the sample down to temperatures as low as 30 K. Heating elements inside and around the cold chamber are used to heat the setup. A Lakeshore Model 336 temperature controller regulates the displex performance and temperature of the exchange gas. The typical time needed to cool down the setup from RT or warm up to RT is 3 h. The whole CryoSEC is mounted on moveable rails (R). It can be detached from the beamline and removed for maintenance while the LiquidSEC sample environment is in use.

Studies of dilute samples in the 2 keV to 6 keV photon energy range require proper attention to vacuum windows manufacturing. Attenuation of the incoming and fluorescent photons should be minimized. However at the same time the windows in the cold sample chamber should withstand a 1 atm differential pressure at 30 K. In addition, the incoming window should not degrade under a high-flux beam. The insulation chamber has exit windows of a similar geometry to the LiquidSEC (see Section 3.4.1). The cold chamber has an exchangeable Al flange with two windows glued with cryogenic epoxy (Stycast 2850FT, Catalyst23LV) to the flange. A 8 mm × 34 mm (H × W) 8 µm-thick Kapton window (W1) serves as an exit window for the fluorescent radiation. The choice of material for the entrance window is more challenging. Even thin 8 µm Kapton will be quickly burned by intense photon beams at energies of 2 keV to 4 keV. Be is used in cryogenic applications but is toxic and becomes fragile at low temperatures. A good alternative is a 1 µm-thick graphenic carbon window (G1, G2) produced by KETEK for RT applications (Huebner *et al.*, 2015 ▸). Our test showed that this window can be also used under cryo-temperatures. This graphenic carbon window has an opening of 7 mm and 90% transmission at 2 keV. We have not observed any degradation after one year of use at the PINK beamline.

During the design phase, emphasis was placed on efficient sample-exchange capabilities because sample changes occur when the cold chamber is opened to atmospheric air. A 1 m-long sample stick with a motorized head is connected to a vacuum flange firmly attached to horizontal rail system (not shown in Fig. 8 ▸), allowing easy sliding of the sample stick from and into the cold chamber. The head of the sample stick is driven by a stepper motor (m1) and can travel 90 mm horizontally. An Attocube cryogenic linear piezo-stage ANPx321 (m2) is attached to the head and provides vertical motion of the sample carrier by ±8 mm. There is an additional heater (H) attached to the back side the piezo-stage. It is used to locally heat the piezo-stage if the cryostat becomes blocked due to ice accumulation. The sample carrier (C) made from PEEK can hold up to seven samples. Samples are loaded into slots via openings on the top of the carrier and forced against the front part of carrier by a Cu–Be spring. Sample loading can be comfortably done in liquid nitrogen or in a glovebox. Afterwards the loaded sample carrier can be contained in a vessel with liquid nitrogen. The sample carrier has a trapezoidal cross-section and can be slid into a machined response groove at the piezo-stage with a single, effortless motion. After venting of the sample chamber with He, the sample-exchange procedure usually takes 15–20 s and can be accomplished by one person. Furthermore, this procedure can be conducted while the cold chamber remains under cryogenic temperature.

A number of XES measurements on S, Fe and Cu complexes using the CryoSEC have already been performed at the PINK beamline and published in the literature (Levin *et al.*, 2020 ▸; Mathe *et al.*, 2021 ▸; Geoghegan *et al.*, 2022 ▸; Gerz *et al.*, 2021 ▸; Hou *et al.*, 2023 ▸; Liu *et al.*, 2023 ▸). Tests conducted with frozen solutions of Fe and Cu containing samples demonstrated that it is feasible to obtain *K*β VtC emission spectra even at concentrations as low as 10 m*M* to 20 m*M*. However, these measurements are time-consuming, typically taking 8 h to 12 h to complete, and are not suitable for samples that are sensitive to radiation. Considering the elevated flux offered by the PINK beamline at lower energies and the augmented fluorescence cross-section of both light elements (*K*β) and transition elements (*L*β), we anticipate achieving lower concentrations ranging from 3 m*M* to 5 m*M* for these elements. At present, this is hindered by the significant contribution of scattering background below 4 keV, which begins to impact the experimental spectrum. Any minor imperfection on the sample surface, presence of gas bubbles or similar factors can introduce individual background patterns and artificial features on the detector. Usually 10% to 30% of the sample surface produces such ‘bad’ spectra. Correction of such irregular background requires additional analysis of the detector images and can be challenging.

### 3.5. Control system

The efficiency and success of an experiment depend on the stability and ease of use of a control system. In addition, the endstation control requires high flexibility and maintainability. Development of the control system was based on three principles. First, use of free and open source software, and avoiding any proprietary software or drivers. Second, development of a friendly framework that enables integration with Python language and HDF5 data format. Third, users and staff should be able to write their own control and acquisition components on demand. After an extended series of tests, a combination of *Experimental Physics and Industrial Control System* (*EPICS*) as a base (https://epics-controls.org), *CS-Studio* and *Phoebus* as a GUI interface (https://www.controlsystemstudio.org), and *PShell* (https://github.com/paulscherrerinstitute/pshell) as an acquisition framework were chosen. The beamline IT infrastructure is based on a Cisco network switch and two Dell PowerEdge servers with multiple network cards. One server uses a series of virtual machines running the different control system services for hardware communication and control. The second server is used for data storage and data analysis. To better organize data exchange, the network is divided into several sub-networks for cameras and detectors, vacuum interlock, hardware communication and guest internet access. Vacuum interlock is managed by a programmable logic controller (PLC). The PLC reads analog signals from multiple vacuum sensors across the beamline and with the combination of vacuum valves it provides control and safe operation of the multiple vacuum components of the beamline. All computers used at the beamline run a long term service (LTS) version of Ubuntu Linux. An electronic logbook based on ELOG is available for users of the beamline to log their experimental information. During operation of the beamline, specific details such as datafile names, sample names, sample motion parameters and detector settings are automatically recorded using Python scripts, which store this information in Google Docs. Each project has its dedicated logbook for organization and data management.

## 4. Results

In this section, the capabilities of the PINK beamline and a comparison with other setups are presented.

**Figure 10:**
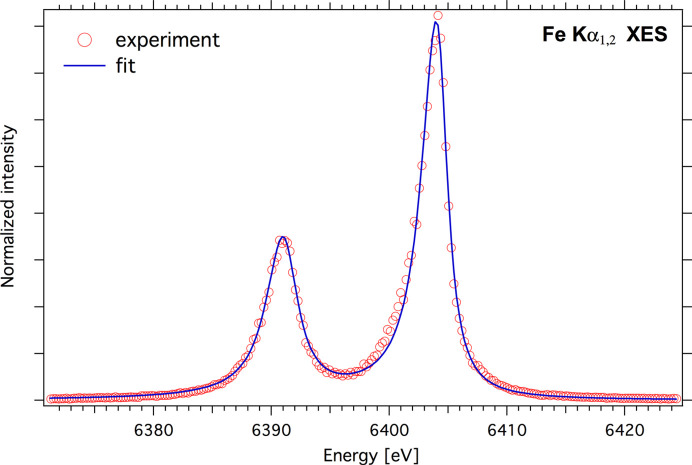
Normalized *K*α_1, 2_ XES spectrum of Fe foil (red open circles) recorded on the atmospheric spectrometer with a fixed-exit direction using a strip bent Si(111) crystal and a best fit to the data (blue line).

A test of the quality of the striped crystal manufacturing was carried out by analysis of the Fe *K*α_1, 2_ spectrum recorded with the atmospheric spectrometer using an Si(333) striped crystal set at a 68° Bragg angle (see Fig. 10 ▸). A ray-tracing simulation gives the resolution for a 1 mm striped crystal with *R* = 250 mm of ∼0.3 eV. The observed emission line yields both the Lorentzian natural linewidth and the Gaussian instrument broadening. We utilized the *lmfit* Python library for fitting purposes. We employed an asymmetric Lorentzian function where the distribution width differs between the left and right slopes. Each emission line was then represented as a convolution of this asymmetric Lorentzian profile with a Gaussian profile. The experimental spectrum was fitted using this newly constructed function. Taking a Lorentzian width of Fe *K*α_1_ and Fe *K*α_2_ lines to 2.55 eV and 3.14 eV, respectively (Hölzer *et al.*, 1997 ▸), the resulting experimental Gaussian broadening, the FWHM of which reflects the spectrometer resolution, was approximately 0.4 eV. This value closely matches that predicted by the ray-tracing value.

**Figure 11:**
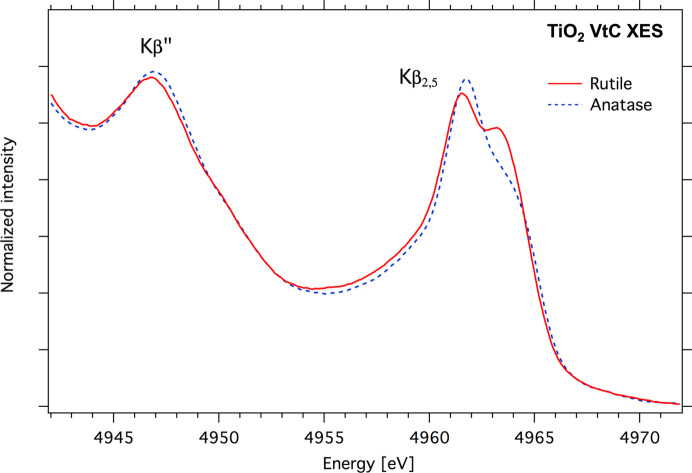
XES VtC spectrum of TiO_2_ in the form of rutile and anatase.

Resolution capabilities of the vacuum von Hamos spectrometer are demonstrated in Fig. 11 ▸. In the work by Swarbrick *et al.* (2010 ▸), it was shown how VtC XES can be applied in the identification of different ligands in Ti complexes. Recent calculations carried out by Miaja-Avila *et al.* (2021 ▸) predicted splitting of the *K*β_2, 5_ emission line on two peaks having different relative intensities. Follow-up measurements done with a microcalorimeter spectrometer could resolve *K*β′ and *K*β_2, 5_ lines but could not resolve the predicted fine structure of the *K*β_2, 5_ line due to the low resolution of the microcalorimeter (∼4 eV).

XES VtC measurements of rutile and anatase powders were performed at the PINK beamline using the vacuum von Hamos spectrometer with an Si(400) striped crystal at Bragg angles of 66.4–69°. In this geometry, the estimated spectrometer resolution was expected to be 0.8 eV or better. The excitation energy was set to 5800 eV. Samples were measured using commercially available powders (Sigma–Aldrich, >99.9% purity). Powders were finely ground, packed into a 1 mm Al cell and sealed from both sides with 30 µm-thick Kapton tape. For the energy calibration procedure, *K*α_1, 2_ emission lines [4952.2 eV and 4944.64 eV (Thompson & Vaughan, 2009 ▸)] of a V powder sample were recorded. The predicted splitting of the *K*β_2, 5_ line is clearly visible in the experimental spectra shown in Fig. 11 ▸.

**Figure 12:**
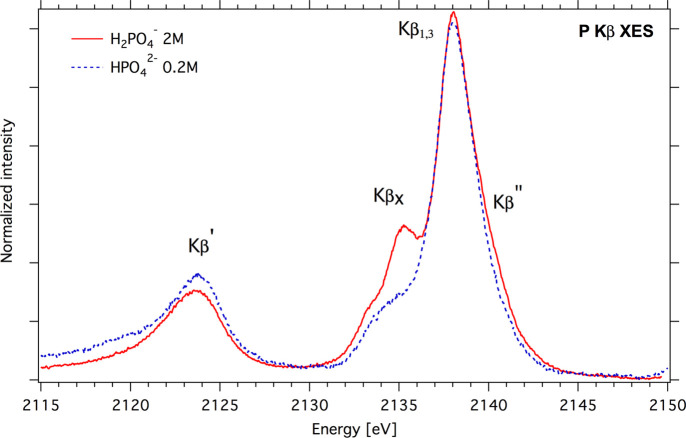
Solution P *K*β spectra of HPO_4_^2−^ and H_2_PO_4_^−^.

The lightest element that is reachable at the PINK beamline is P. Solid spectra of orthophosphates NaH_2_PO_4_ and Na_2_HPO_4_ were measured in earlier work by Petric *et al.* (2015 ▸). In Fig. 12 ▸, P *K*β solution spectra of NaH_2_PO_4_ and Na_2_HPO_4_ are shown, collected at RT using a liquid cell installed in the LiquidSEC sample chamber (Mathe *et al.*, 2021 ▸). The data were collected at an excitation energy of 4 keV (incoming flux 2 × 10^13^ photons s^−1^) using the vacuum spectrometer. An Si(111) crystal accepted Bragg angles of 69.3° to 66.7° with the corresponding energy range 2110 eV to 2150 eV. The analyzer resolution was estimated to be ∼0.3 eV. For the energy calibration procedure, a powder NaH_2_PO_4_ standard was chosen. The *K*β XES NaH_2_PO_4_ spectrum was fitted with four Voigt profiles that correspond to 2139.5 eV, 2137.9 eV, 2135.3 eV and 2123.4 eV (Petric *et al.*, 2015 ▸).

**Figure 13:**
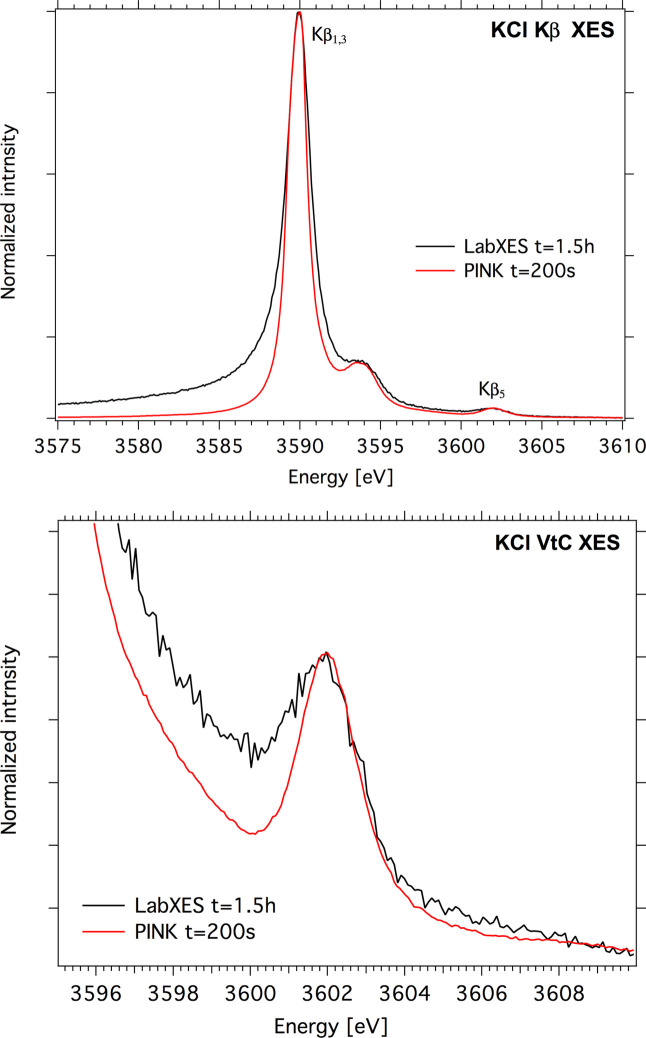
Comparison of K *K*β spectra of a KCl powder sample measured at the PINK beamline and LabXES laboratory setup at CEC MPI; the measurement times were 200 s and 1.5 h, respectively.

An example *K*β spectrum of a KCl powder sample measured at the PINK beamline with a vacuum spectrometer using an Si(220) strip bent crystal set at 64° Bragg angle is shown in Fig. 13 ▸. The spectrum has three well resolved peaks. The main line *K*β_1, 3_ at 3589.9 eV is attributed to the 3*p*–1*s* transition. The approximately 5× lower intensity *K*β_*x*_ satellite peak at ∼3593.5 eV can be explained by double ionization of the K 3*p* level. A low-intensity signal at 3602 eV is assigned to the *K*β_5_ VtC transition (Deslattes, 1964 ▸; Best, 1971 ▸). A more detailed analysis of VtC spectra of K salts will be published soon. For comparison, a KCl spectrum measured at the LabXES laboratory setup using a metal jet X-ray source and a von Hamos spectrometer with a ring HAPG crystal is presented.

**Figure 14:**
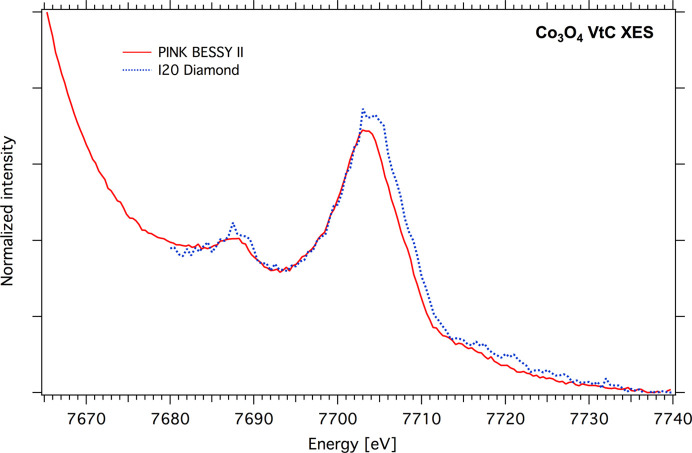
Comparison of VtC XES of Co_3_O_4_ powder samples measured at the Diamond I20 beamline (blue) and PINK setup (red); the measurement time was 15 min.

Scanning spectrometers based on Johann geometry are traditionally characterized with better signal-to-noise ratio than von Hamos spectrometers. Indeed, the detected photon flux per energy unit is usually much lower for long-radius von Hamos spectrometers, but shortening the distance between the sample and the dispersive crystal should improve the von Hamos spectrometer performance. Fig. 14 ▸ presents the Co *K*β VtC spectrum of Co_3_O_4_ powder recorded at the I20 beamline (Diamond Light Source) with a Johann emission spectrometer based on a 1 m Rowland circle and the PINK short-radius atmospheric spectrometer [striped Si(620) crystal, *R* = 250 mm]. The spectra demonstrate comparable signal-to-noise ratios and resolutions well suited for VtC XES experiments. Both spectra have the same incoming photon flux of ∼10^13^ photons s^−1^ and total measurement time *t* = 15 min; but in the case of the Johann spectrometer, the detector collection time is smaller due to the necessity to scan the analyzer crystal. An additional bonus of the PINK von Hamos spectrometer is a detected energy range of ∼350 eV, thus both the *K*β_2,5_ and the *K*β_1,3_ main lines were recorded in the same time. During a VtC spectra analysis, the main line can be useful for normalization procedures.

## 5. Conclusions

We have described the PINK beamline and its specialized endstation optimized for non-resonant XES studies within the tender X-ray energy range. Our primary focus is investigating VtC photon-hungry emission lines. Photon flux ranges from 10^13^ photons s^−1^ to 10^14^ photons s^−1^ covering the energy range 2.1 keV to 9.5 keV. This range encompasses the *K*α and *K*β lines of elements from P to Cu, as well as the *L*α and *L*β lines of second row transition metals including Mo, Ru, Rh, Pd *etc*.

The endstation is equipped with two von Hamos dispersive spectrometers, finely tuned for the energy ranges 2 keV to 6 keV and 6 keV to 9.5 keV. Users have access to two sample environment chambers: the LiquidSEC chamber for experiments with solid and liquid samples at RT, including the option for electrochemical experiments using an electrochemical cell under vacuum or an He atmosphere; the CryoSEC chamber is designed for frozen samples and operates at 30 K, accommodating up to seven samples with a fast sample-exchange system.

By combining high flux, quick sample scanning, cryogenic temperatures and the capabilities of a dispersive von Hamos spectrometer to record the full spectrum in a single shot, we can obtain radiation-damage-free VtC spectra for sensitive and dilute complexes. We have provided case studies for P, Ru, Ti and Co complexes to showcase the beamline capabilities. The PINK beamline is fully operational and open to external users. In future work, the current setup will be expanded to include resonant XES and VtC-enhanced XAS techniques.

## 6. Data availability

The data used for the figure production are available at https://doi.org/10.17617/3.4ZB7ZR.

## Figures and Tables

**Figure 9:**
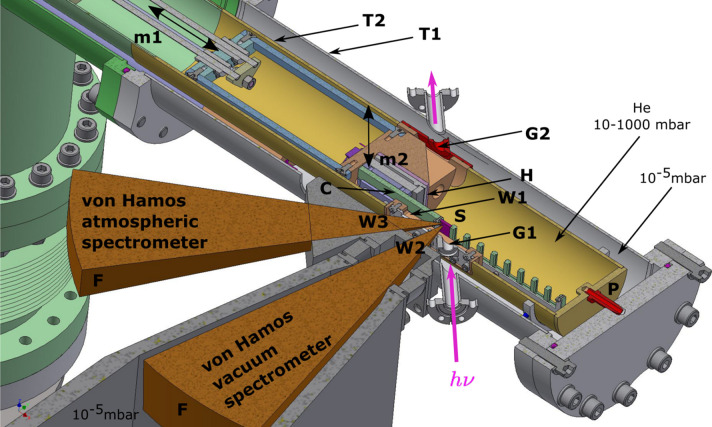
Scheme of a part of the CryoSEC connected to the beamline and the spectrometers. T1 – insulation tube; T2 – cold copper tube; P – pin centering the inner copper tube; C – sample carrier; S – irradiated sample; m1 – linear stage providing horizontal motion of a sample carrier (90 mm); m2 – vertical linear piezo stage (±8 mm); H – heater for the piezo-stage; G1 and G2 – graphenic carbon 1 µm-thick windows; Kapton vacuum windows: W1 – 13 µm, W2 – 8 µm, W3 – 25 µm; F – fluorescent light emitted from the sample in the direction of the spectrometers.

**Table 1:** Energy and bandwidth of the multilayer monochromator

Mirror	1			2			3		
Stripe	1	2	3	1	2	3	1	2	3
Energy (keV)	2.3	3.0	4.0	5.2	5.8	6.8	7.3	8.0	9.5
FWHM (eV)	155	117	80	65	115	90	123	110	100

**Table 2:** Working specification of the atmospheric and vacuum von Hamos spectrometers installed at the PINK beamline All measured samples were in powder form unless indicated by an asterisk.

	Atmospheric VH#1	Vacuum VH#2
Crystal radius	*R = *250 (mm)	
Available crystals	Bent: Si(100), Si(110), Si(111), Si(311), quartz(1012)	
	Strip bent: Si(100), Si(1110), Si(111), Ge(100), Ge(110), Ge(111), Si(310)	
Crystal dimensions	Quartz(1012): 25 mm × 100 mm; Si(311): 30 mm × 100 mm; other: 50 mm × 100 mm	
Bragg angle	45–82° (possible)	Chamber 1: 59–81°
	60–82° (in use)	Chamber 2: 49–61° (S, Mo)
Energy range	6–10 eV	2–6 eV
Energy window	300–500 eV	20–80 eV
Energy resolution	0.4–1 eV	
Solid angle	(0.9–1.5) × 10^−4^ sr eV^−1^	
Detector	Mythen2 1K X (8 mm × 64 mm)	CCD GreatEyes (6 mm × 26 mm)
	Eiger2 R 500K (40 mm × 80 mm)	
Typical acquisition time	Co VtC (Co_2_O_3_): 20 min	P *K*β (KH_2_PO_4_): 3 min
	Fe VtC (FeCl_3_ 10 m*M*): 12 h^*^	P *K*β (Na_3_ADP, 30 m*M*): 40 min^*^
	Cu VtC (CuCl): 40 min	K VtC (KCl): 5 min
		Ru *L*β_2_ {[Ru(tacn)_2_](PF_6_)_2_}: 1 h
		Ru *L*γ_1_ [Ru(NH_3_)_6_Cl_3_]: 15 min

## References

[bb1] Abraham, B., Nowak, S., Weninger, C., Armenta, R., Defever, J., Day, D., Carini, G., Nakahara, K., Gallo, A., Nelson, S., Nordlund, D., Kroll, T., Hunter, M. S., van Driel, T., Zhu, D., Weng, T.-C., Alonso-Mori, R. & Sokaras, D. (2019). *J. Synchrotron Rad.* **26**, 629–634. 10.1107/S1600577519002431 PMC6510194 31074425

[bb2] Abramson, J. E., Holden, W. M., Rivera-Maldonado, R. A., Velian, A., Cossairt, B. M. & Seidler, G. T. (2023). *J. Anal. At. Spectrom.* **38**, 1125–1134.

[bb3] Alonso-Mori, R., Kern, J., Sokaras, D., Weng, T.-C., Nordlund, D., Tran, R., Montanez, P., Delor, J., Yachandra, V. K., Yano, J. & Bergmann, U. (2012). *Rev. Sci. Instrum.* **83**, 073114. 10.1063/1.4737630 PMC3422323 22852678

[bb4] Bauer, M. (2014). *Phys. Chem. Chem. Phys.* **16**, 13827–13837. 10.1039/c4cp00904e 24905791

[bb5] Best, P. E. (1971). *Phys. Rev. B*, **3**, 4377–4382.

[bb7] Błachucki, W., Czapla-Masztafiak, J., Sá, J. & Szlachetko, J. (2019). *J. Anal. At. Spectrom.* **34**, 1409–1415.

[bb6] Butorin, S. M., Kvashnina, K. O., Klintenberg, M., Kavčič, M., Žitnik, M., Bučar, K., Gougeon, P., Gall, P., Candolfi, C. & Lenoir, B. (2018). *ACS Appl. Energy Mater.* **1**, 4032–4039.

[bb8] Canton, S. E., Biednov, M., Pápai, M., Lima, F. A., Choi, T., Otte, F., Jiang, Y., Frankenberger, P., Knoll, M., Zalden, P., Gawelda, W., Rahaman, A., Møller, K. B., Milne, C., Gosztola, D. J., Zheng, K., Retegan, M. & Khakhulin, D. (2023). *Adv. Sci.* **10**, 2206880. 10.1002/advs.202206880 PMC10375196 37196414

[bb9] Castillo, R. G., Banerjee, R., Allpress, C. J., Rohde, G. T., Bill, E., Que, L., Lipscomb, J. D. & DeBeer, S. (2017). *J. Am. Chem. Soc.* **139**, 18024–18033. 10.1021/jacs.7b09560 PMC5729100 29136468

[bb10] Castillo, R. G., Hahn, A. W., Van Kuiken, B. E., Henthorn, J. T., McGale, J. & DeBeer, S. (2021). *Angew. Chem. Int. Ed.* **60**, 10112–10121. 10.1002/anie.202015669 PMC8252016 33497500

[bb11] Castillo, R. G., Henthorn, J. T., McGale, J., Maganas, D. & DeBeer, S. (2020). *Angew. Chem. Int. Ed.* **59**, 12965–12975. 10.1002/anie.202003621 PMC7496169 32363668

[bb12] Cutsail, G. E. III, Banerjee, R., Zhou, A., Que, L., Lipscomb, J. D. & DeBeer, S. (2018). *J. Am. Chem. Soc.* **140**, 16807–16820. 10.1021/jacs.8b10313 PMC6470014 30398343

[bb14] Cutsail, G. E. III & DeBeer, S. (2022). *ACS Catal.* **12**, 5864–5886.

[bb13] Cutsail, G. E. III, Gagnon, N. L., Spaeth, A. D., Tolman, W. B. & DeBeer, S. (2019). *Angew. Chem. Int. Ed.* **58**, 9114–9119. 10.1002/anie.201903749 PMC6615971 30994976

[bb15] Deslattes, R. D. (1964). *Phys. Rev.* **133**, A390–A398.

[bb16] Follath, R., Hävecker, M., Reichardt, G., Lips, K., Bahrdt, J., Schäfers, F. & Schmid, P. (2013). *J. Phys. Conf. Ser.* **425**, 212003.

[bb17] Geoghegan, B. L., Liu, Y., Peredkov, S., Dechert, S., Meyer, F., DeBeer, S. & Cutsail, G. E. (2022). *J. Am. Chem. Soc.* **144**, 2520–2534. 10.1021/jacs.1c09505 PMC8855422 35050605

[bb18] Gerz, I., Jannuzzi, S. A. V., Hylland, K. T., Negri, C., Wragg, D. S., Øien–Ødegaard, S., Tilset, M., Olsbye, U., DeBeer, S. & Amedjkouh, M. (2021). *Eur. J. Inorg. Chem.* **2021**, 4762–4775. 10.1002/ejic.202100722 PMC9298233 35874966

[bb19] Glatzel, P., Weng, T.-C., Kvashnina, K., Swarbrick, J., Sikora, M., Gallo, E., Smolentsev, N. & Mori, R. A. (2013). *J. Electron Spectrosc. Relat. Phenom.* **188**, 17–25.

[bb20] Groot, F. de (2001). *Chem. Rev.* **101**, 1779–1808. 10.1021/cr9900681 11709999

[bb21] Hall, E. R., Pollock, C. J., Bendix, J., Collins, T. J., Glatzel, P. & DeBeer, S. (2014). *J. Am. Chem. Soc.* **136**, 10076–10084. 10.1021/ja504206y 24946007

[bb28] Hämäläinen, K., Siddons, D. P., Hastings, J. B. & Berman, L. E. (1991). *Phys. Rev. Lett.* **67**, 2850–2853. 10.1103/PhysRevLett.67.2850 10044570

[bb58] Hámos, L. von (1933). *Ann. Phys.* **409**, 716–724.

[bb22] Hendel, S., Schäfers, F., Hävecker, M., Reichardt, G., Scheer, M., Bahrdt, J. & Lips, K. (2016). *AIP Conf. Proc.* **1741**, 030038

[bb23] Holden, W. M., Hoidn, O. R., Ditter, A. S., Seidler, G. T., Kas, J., Stein, J. L., Cossairt, B. M., Kozimor, S. A., Guo, J., Ye, Y., Marcus, M. A. & Fakra, S. (2017). *Rev. Sci. Instrum.* **88**, 073904. 10.1063/1.4994739 28764488

[bb24] Holden, W. M., Seidler, G. T. & Cheah, S. (2018). *J. Phys. Chem. A*, **122**, 5153–5161. 10.1021/acs.jpca.8b02816 29781610

[bb29] Hölzer, G., Fritsch, M., Deutsch, M., Härtwig, J. & Förster, E. (1997). *Phys. Rev. A*, **56**, 4554–4568.

[bb25] Hoszowska, J., Dousse, J.-C., Kern, J. & Rhême, C. (1996). *Nucl. Instrum. Methods Phys. Res. A*, **376**, 129–138.

[bb26] Hou, K., Börgel, J., Jiang, H. Z. H., SantaLucia, D. J., Kwon, H., Zhuang, H., Chakarawet, K., Rohde, R. C., Taylor, J. W., Dun, C., Paley, M. V., Turkiewicz, A. B., Park, J. G., Mao, H., Zhu, Z., Alp, E. E., Zhao, J., Hu, M. Y., Lavina, B., Peredkov, S., Lv, X., Oktawiec, J., Meihaus, K. R., Pantazis, D. A., Vandone, M., Colombo, V., Bill, E., Urban, J. J., Britt, R. D., Grandjean, F., Long, G. J., DeBeer, S., Neese, F., Reimer, J. A. & Long, J. R. (2023). *Science*, **382**, 547–553. 10.1126/science.add7417 37917685

[bb27] Huebner, S., Miyakawa, N., Kapser, S., Pahlke, A. & Kreupl, F. (2015). *IEEE Trans. Nucl. Sci.* **62**, 588–593.

[bb30] Kalinko, A., Caliebe, W. A., Schoch, R. & Bauer, M. (2020). *J. Synchrotron Rad.* **27**, 31–36. 10.1107/S1600577519013638 PMC6927517 31868733

[bb31] Klementiev, K. & Chernikov, R. (2014). *Proc. SPIE*, **9209**, 92090A.

[bb32] Kowalska, J. K., Lima, F. A., Pollock, C. J., Rees, J. A. & DeBeer, S. (2016). *Isr. J. Chem.* **56**, 803–815.

[bb33] Lancaster, K. M., Roemelt, M., Ettenhuber, P., Hu, Y., Ribbe, M. W., Neese, F., Bergmann, U. & DeBeer, S. (2011). *Science*, **334**, 974–977. 10.1126/science.1206445 PMC3800678 22096198

[bb34] Levin, N., Peredkov, S., Weyhermüller, T., Rüdiger, O., Pereira, N. B., Grötzsch, D., Kalinko, A. & DeBeer, S. (2020). *Inorg. Chem.* **59**, 8272–8283. 10.1021/acs.inorgchem.0c00663 PMC7298721 32390417

[bb35] Lima, F. A., Bjornsson, R., Weyhermüller, T., Chandrasekaran, P., Glatzel, P., Neese, F. & DeBeer, S. (2013). *Phys. Chem. Chem. Phys.* **15**, 20911. 10.1039/c3cp53133c 24197060

[bb36] Liu, Y., Chatterjee, S., Cutsail, G. E. III, Peredkov, S., Gupta, S. K., Dechert, S., DeBeer, S. & Meyer, F. (2023). *J. Am. Chem. Soc.* **145**, 18477–18486. 10.1021/jacs.3c04893 PMC10450684 37565682

[bb37] Liu, Z., Yuge, K. & Kawai, J. (2004). *At. Spectrosc.* **59**, 93–99.

[bb38] Maganas, D., DeBeer, S. & Neese, F. (2017). *Inorg. Chem.* **56**, 11819–11836. 10.1021/acs.inorgchem.7b01810 PMC5692824 28920680

[bb39] Malzer, W., Grötzsch, D., Gnewkow, R., Schlesiger, C., Kowalewski, F., Van Kuiken, B., DeBeer, S. & Kanngießer, B. (2018). *Rev. Sci. Instrum.* **89**, 113111. 10.1063/1.5035171 30501328

[bb40] Mathe, Z., McCubbin Stepanic, O., Peredkov, S. & DeBeer, S. (2021). *Chem. Sci.* **12**, 7888–7901. 10.1039/d1sc01266e PMC8188515 34168842

[bb41] Meents, A., Gutmann, S., Wagner, A. & Schulze-Briese, C. (2010). *Proc. Natl Acad. Sci. USA*, **107**, 1094–1099. 10.1073/pnas.0905481107 PMC2798883 20080548

[bb42] Miaja–Avila, L., O’Neil, G. C., Joe, Y. I., Morgan, K. M., Fowler, J. W., Doriese, W. B., Ganly, B., Lu, D., Ravel, B., Swetz, D. S. & Ullom, J. N. (2021). *X-ray Spectrom.* **50**, 9–20. 10.1002/xrs.3183 PMC11465483 39391149

[bb43] Mori, R. A., Paris, E., Giuli, G., Eeckhout, S. G., Kavčič, M., Žitnik, M., Bučar, K., Pettersson, L. G. M. & Glatzel, P. (2010). *Inorg. Chem.* **49**, 6468–6473. 10.1021/ic100304z 20553025

[bb44] Petric, M., Bohinc, R., Bučar, K., Žitnik, M., Szlachetko, J. & Kavčič, M. (2015). *Anal. Chem.* **87**, 5632–5639. 10.1021/acs.analchem.5b00782 25927339

[bb45] Petric, M. & Kavčič, M. (2016). *J. Anal. At. Spectrom.* **31**, 450–457.

[bb46] Pollock, C. J. & DeBeer, S. (2015). *Acc. Chem. Res.* **48**, 2967–2975. 10.1021/acs.accounts.5b00309 26401686

[bb47] Pollock, C. J., Grubel, K., Holland, P. L. & DeBeer, S. (2013). *J. Am. Chem. Soc.* **135**, 11803–11808. 10.1021/ja3116247 PMC3811143 23862983

[bb48] Qureshi, M., Nowak, S. H., Vogt, L. I., Cotelesage, J. J. H., Dolgova, N. V., Sharifi, S., Kroll, T., Nordlund, D., Alonso-Mori, R., Weng, T.-C., Pickering, I. J., George, G. N. & Sokaras, D. (2021). *Phys. Chem. Chem. Phys.* **23**, 4500–4508. 10.1039/d0cp05323f 33355326

[bb49] Rehanek, J., Milne, C. J., Szlachetko, J., Czapla-Masztafiak, J., Schneider, J., Huthwelker, T., Borca, C. N., Wetter, R., Patthey, L. & Juranić, P. (2018). *J. Synchrotron Rad.* **25**, 16–19. 10.1107/S1600577517012796 PMC5741116 29271745

[bb50] Rovezzi, M., Harris, A., Detlefs, B., Bohdan, T., Svyazhin, A., Santambrogio, A., Degler, D., Baran, R., Reynier, B., Noguera Crespo, P., Heyman, C., Van Der Kleij, H.-P., Van Vaerenbergh, P., Marion, P., Vitoux, H., Lapras, C., Verbeni, R., Kocsis, M. M., Manceau, A. & Glatzel, P. (2020). *J. Synchrotron Rad.* **27**, 813–826. 10.1107/S160057752000243X PMC7285681 32381786

[bb59] Schooneveld, M. M. van & DeBeer, S. (2015). *J. Electron Spectrosc. Relat. Phenom.* **198**, 31–56.

[bb51] Seidler, G. T., Mortensen, D. R., Remesnik, A. J., Pacold, J. I., Ball, N. A., Barry, N., Styczinski, M. & Hoidn, O. R. (2014). *Rev. Sci. Instrum.* **85**, 113906. 10.1063/1.4901599 25430123

[bb52] Swarbrick, J. C., Kvashnin, Y., Schulte, K., Seenivasan, K., Lamberti, C. & Glatzel, P. (2010). *Inorg. Chem.* **49**, 8323–8332. 10.1021/ic100755t 20831281

[bb53] Szlachetko, J., Nachtegaal, M., de Boni, E., Willimann, M., Safonova, O., Sa, J., Smolentsev, G., Szlachetko, M., van Bokhoven, J. A., Dousse, J.-C., Hoszowska, J., Kayser, Y., Jagodzinski, P., Bergamaschi, A., Schmitt, B., David, C. & Lücke, A. (2012). *Rev. Sci. Instrum.* **83**, 103105. 10.1063/1.4756691 23126749

[bb54] Szlachetko, J., Nachtegaal, M., Grolimund, D., Knopp, G., Peredkov, S., Czapla–Masztafiak, J. & Milne, C. (2017). *Appl. Sci.* **7**, 899.

[bb55] Szlachetko, J., Sá, J., Safonova, O., Smolentsev, G., Szlachetko, M., van Bokhoven, J. & Nachtegaal, M. (2013). *J. Electron Spectrosc. Relat. Phenom.* **188**, 161–165.

[bb56] Thompson, A. & Vaughan, D. (2009). *X-ray Data Booklet.* Center for X-ray Optics and Advanced Light Source, Lawrence Berkeley National Laboratory, Berkeley, USA.

[bb57] Tono, K., Kudo, T., Yabashi, M., Tachibana, T., Feng, Y., Fritz, D., Hastings, J. & Ishikawa, T. (2011). *Rev. Sci. Instrum.* **82**, 023108. 10.1063/1.3549133 21361574

[bb60] Vane, C. R., Smith, M. S. & Raman, S. (1988). *A Versatile, High-Efficient, High-Resolution von Hamos Bragg Crystal X-ray Spectrometer.* Oak Rodge National Laboratory, Oak Ridge, Tennessee, USA.

[bb61] Zimmermann, P., Peredkov, S., Abdala, P. M., DeBeer, S., Tromp, M., Müller, C. & van Bokhoven, J. A. (2020). *Coord. Chem. Rev.* **423**, 213466.

